# Effectiveness, survival and safety of guselkumab attending to basal characteristics in moderate-to-severe psoriatic patients: a cohort study

**DOI:** 10.12688/f1000research.122945.2

**Published:** 2022-11-07

**Authors:** Ricardo Ruiz-Villaverde, Lourdes Rodriguez-Fernandez-Freire, Jose C. Armario-Hita, Amalia Pérez-Gil, Fiorella Vasquez Chinchay, Manuel Galán-Gutiérrez

**Affiliations:** 1 Dermatology Department, Hospital Universitario San Cecilio, Granada, 18016, Spain; 2 Dermatology Department, Hospital Universitario Virgen del Rocio, Sevilla, 41013, Spain; 3 Dermatology Department, Hospital Universitario Puerto Real, Cádiz, 11510, Spain; 4 Dermatology Department, Hospital Universitario Virgen de Valme, Sevilla, 41014, Spain; 5 Dermatology Department, Hospital Quirón Salud Sagrado Corazón, Sevilla, 41013, Spain; 6 Dermatology Department, Hospital Universitario Reina Sofía, Córdoba, 14004, Spain

**Keywords:** Guselkumab, Psoriasis, Effectiveness, Survival, Safety, Demographic characteristics

## Abstract

**Background: **Psoriasis is a chronic inflammatory disease which can impact quality of life. In the past decade multiple biologic treatments have been released with encouraging results. Guselkumab is a monoclonal antibody targeting IL-23p19. Multiple randomized clinical trials have demonstrated its efficacy in psoriasis, but response differences among patient subpopulations have not been extensively reported. Furthermore, patients in real life are often non-eligible for clinical trials and their responses may differ from pivotal studies.

**Methods**: This is a retrospective, observational study of real clinical practice of patients receiving guselkumab treatment in Spain. Patients treated with guselkumab were included between February 2019 to December 2021. This study evaluates the potential differential effect of baseline demographic and disease characteristics on therapeutic responses to guselkumab. We measured effectiveness and survival by the psoriasis area and severity index, the dermatology life quality index as well as Kaplan meier curves, respectively. Categorical and quantitative variables are reported with frequencies, and with mean and standard deviation, respectively. Differences between groups in psoriasis area and severity index and dermatology life quality index, were calculated using a mixed-effects analysis. Survival was calculated using Kaplan meier curves and log-rank tests.

**Results**: A total of 87 patients were included. In this study, our objective was to evaluate the effectiveness, safety and survival of guselkumab attending to demographic characteristics. No differences in psoriasis area and severity index or dermatology life quality index baseline values or therapeutic responses were noted at 52 weeks of follow-up among all the subgroups analysed (age, sex, psoriasis duration, body mass index, and comorbidities). A difference in drug survival was only seen between gender groups.

**Conclusions: **Our research has demonstrated the consistency of guselkumab effectiveness across patient subgroups. No baseline features affected the effectiveness or drug survival of guselkumab, except for lower drug survival in female patients.

## Introduction

Psoriasis (PSO) is a chronic inflammatory disease characterized by skin lesions that can be painful, disabling, disfiguring, and thereby negatively impact the patients’ quality of life. ^1^ It has an impact on the general quality of life (as measured by the dermatology life quality index (DLQI)) and the pruritus that may result (visual analog scale (VAS) pruritus), as well as the psychological sphere, work productivity, and personal and family relationships. ^2^ PSO affects between 1.5-3% of the general population in Europe, with a similar prevalence in men and women. ^1^^–^^3^

Treatment of moderate to severe PSO has radically changed in the past decade; patients have benefited from translational research leading to development of multiple biologic agents such as anti-IL-17 ^3^^,^^4^ and anti-IL23p19. ^5^^,^^6^ Our current understanding of the pathophysiology of PSO is characterized by a cytokine disbalance with predominant involvement of the interleukin (IL)-23/Th-17 axis, as well as keratinocyte hyperproliferation and immune activation. ^1^^–^^3^

IL-23 produced by dendritic cells and keratinocytes in PSO plaques promotes the differentiation, expansion and maintenance of Th17 as well as the production of the corresponding cytokines such as IL-17A, IL-17F, TNFα and IL-22. ^7^ The role of IL-23 as “master regulator” of inflammation in PSO has justified the development of selective IL-23p19 inhibitors and was confirmed by their therapeutic success. ^2^^–^^4^

Guselkumab (GUS) is a fully human anti-IL-23 immunoglobulin-G1-lambda monoclonal antibody, that binds the p19 subunit of interleukin 23. ^5^ It has been currently approved by the United States Food and Drug Administration (FDA) and the European Medicines Agency (EMA) for the treatment of adult patients with moderate-to-severe PSO who are candidates for systemic therapy. ^2^ The EMA has also approved the use of GUS for treatment of active psoriatic arthritis in adult patients who have had an inadequate response or who have been intolerant to a prior disease-modifying antirheumatic drug therapy. ^2^^,^^6^ Three phase-III, randomized, double-blind, placebo-controlled clinical trials have proved the efficacy and safety of GUS in PSO treatment. ^7^^–^^9^ VOYAGE 1 and VOYAGE 2 randomized clinical trials compared GUS to adalimumab showing its superiority at 16 weeks and increasing and maintaining these results over time. ^7^^,^^8^ The NAVIGATE trial, a phase-III, multicenter, randomised, double-blind trial that compared the efficacy of the switching from UST to GUS in patients presenting an inadequate response (IGA≥2) after 16 weeks open-label treatment with the anti-IL12/23. ^8^ Furthermore, GUS treatment has been compared to other biologics, such as secukinumab, and the CLEAR head-to-head trial proved the superiority of this anti-IL23p19 antagonist. ^3^

The response of various subpopulations of patients — defined by baseline demographic features including weight, PSO disease characteristics and previous PSO treatments — has been assessed using pooled data from the VOYAGE 1 and VOYAGE 2 trials. ^10^^,^^11^ GUS has proved superiority to placebo and adalimumab at week 16 and week 24 respectively in all subpopulations except in the African American population, which included few patients. ^10^^,^^11^ There is scarce evidence on the behaviour of GUS in different profile of patients. Patients in real life are often non-eligible for clinical trials, and their response, as regards efficacy and safety, may differ from those in pivotal studies. Thus, findings in clinical trials may not be extrapolatable to real life practice. ^9^

Accordingly, we have performed a retrospective, longitudinal, observational study of real clinical practice of patients receiving treatment with GUS 100 mg subcutaneously every eight weeks in six tertiary hospitals in Spain in order to evaluate the potential differential effect of baseline demographic and disease characteristics on therapeutic response to this biologic agent.

## Methods

This is a retrospective, observational study of real clinical practice of patients receiving GUS treatment in Spain. It included patients from six different dermatological centers in Spain who started treatment with guselkumab between February 2019 to December 2020. The information of the patients was collected retrospectively from clinical records. The inclusion criteria were patients with moderate-to-severe psoriasis on guselkumab treatment for their psoriasis. Patients with other inflammatory diseases were excluded from the study. We use PASI and DLQI as variables to measure the effectiveness of GUS between subgroup of patients. Survival was measured by Kaplan-Meyer curves and safety by the reporting of adverse events. As a limitation of the study, we did not apply any method to avoid bias. Categorical and quantitative variables are reported with frequencies (%), and with mean and standard deviation (SD), respectively. Differences between groups in PASI and DLQI, were calculated using a mixed-effects analysis. Survival was calculated using Kaplan-Meyer curves and log-rank tests.

### Patients

A total of 87 patients with moderate-to-severe plaque PSO (PASI >10, BSA>10 +/- DLQI 10) treated with GUS were included in this retrospective observational study. This cross-sectional analysis includes information of patients who started treatment with GUS between February 2019 to December 2020. A total of six tertiary hospitals in Andalusia (Spain) participated in this study: Hospital Universitario San Cecilio, (Granada, Spain), Hospital Universitario Virgen del Rocio, (Sevilla, Spain); Hospital Universitario Puerto Real, (Puerto Real, Cádiz, Spain); Hospital Universitario Virgen de Valme, (Sevilla, Spain); Hospital Quirón Salud Sagrado Corazón (Sevilla, Spain), Hospital Universitario Reina Sofia (Córdoba, Spain). Information from patients was collected retrospectively from clinical records. This study has been approved by Ethics Committee of Hospital Universitario San Cecilio (DER-HUSC-2020-004). Before inclusion into the study, patients gave their written informed consent.

The inclusion criteria used for this study were: 1) adult moderate-to-severe plaque PSO patients; 2) PSO diagnosis from over 1 year ago; 3) patients on GUS treatment 100 mg subcutaneous (at week 0 and 4, followed by a maintenance dose every 8 weeks; other posology were also included). The exclusion criteria were: 1) presence of other inflammatory diseases such as rheumatoid arthritis, Crohn disease, ulcerative colitis and/or ankylosing spondylitis. Missing data at different timepoints was due in part to COVID19 situation, where some patients refused to attend to the hospital for medical follow-up.

### Outcome measures

**Figure 1.**
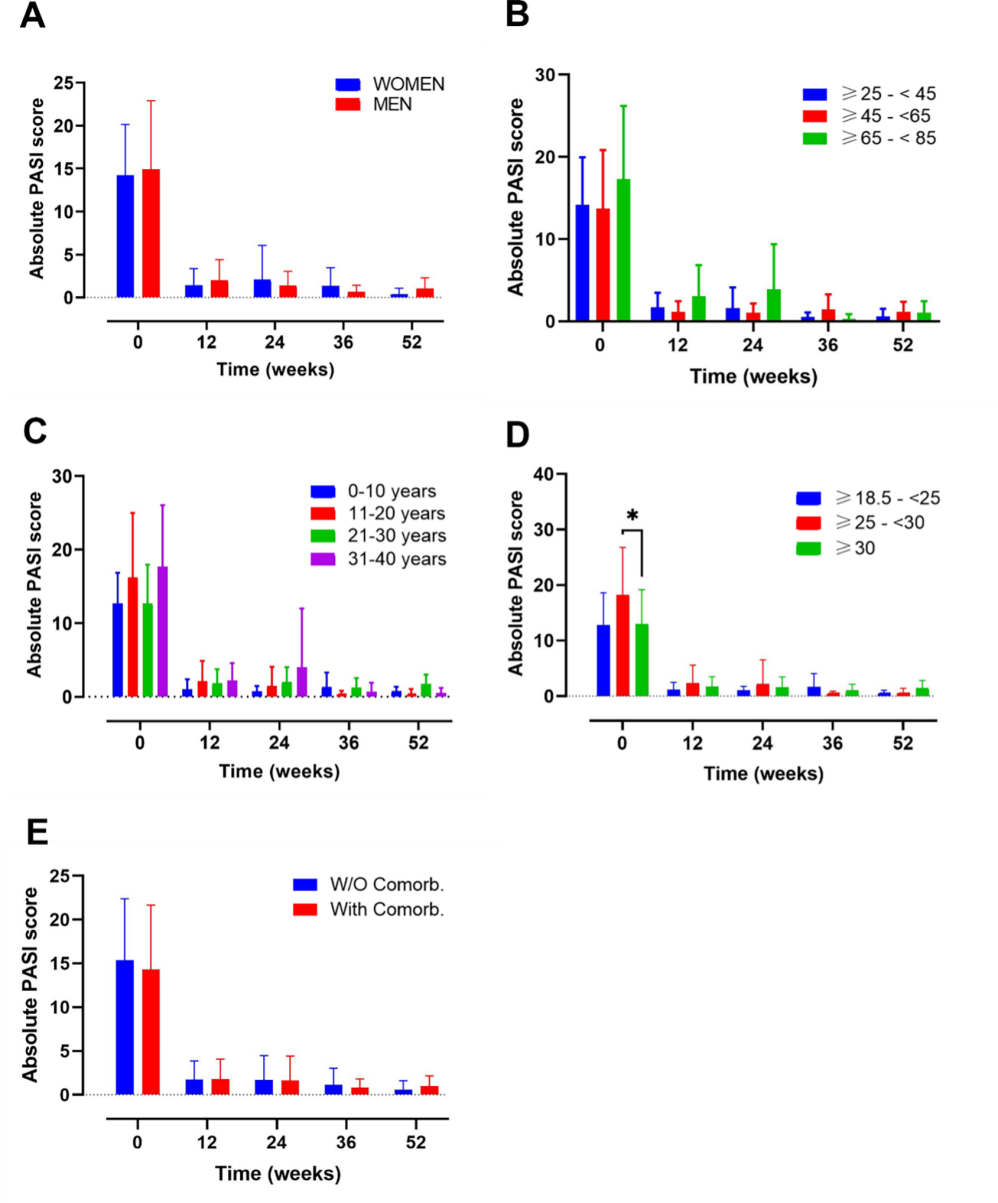
Evolution of absolute psoriasis area and severity index (PASI) score over time in different subgroups of patients during treatment with guselkumab. Evolution of absolute PASI score in A) female vs male; B) ranges of age; C) year of psoriasis evolution; D) ranges of body mass index and E) patients with or without comorbidities. Mixed-effects analysis, *, p<0.05. PASI, psoriasis area and severity index.

**Figure 2.**
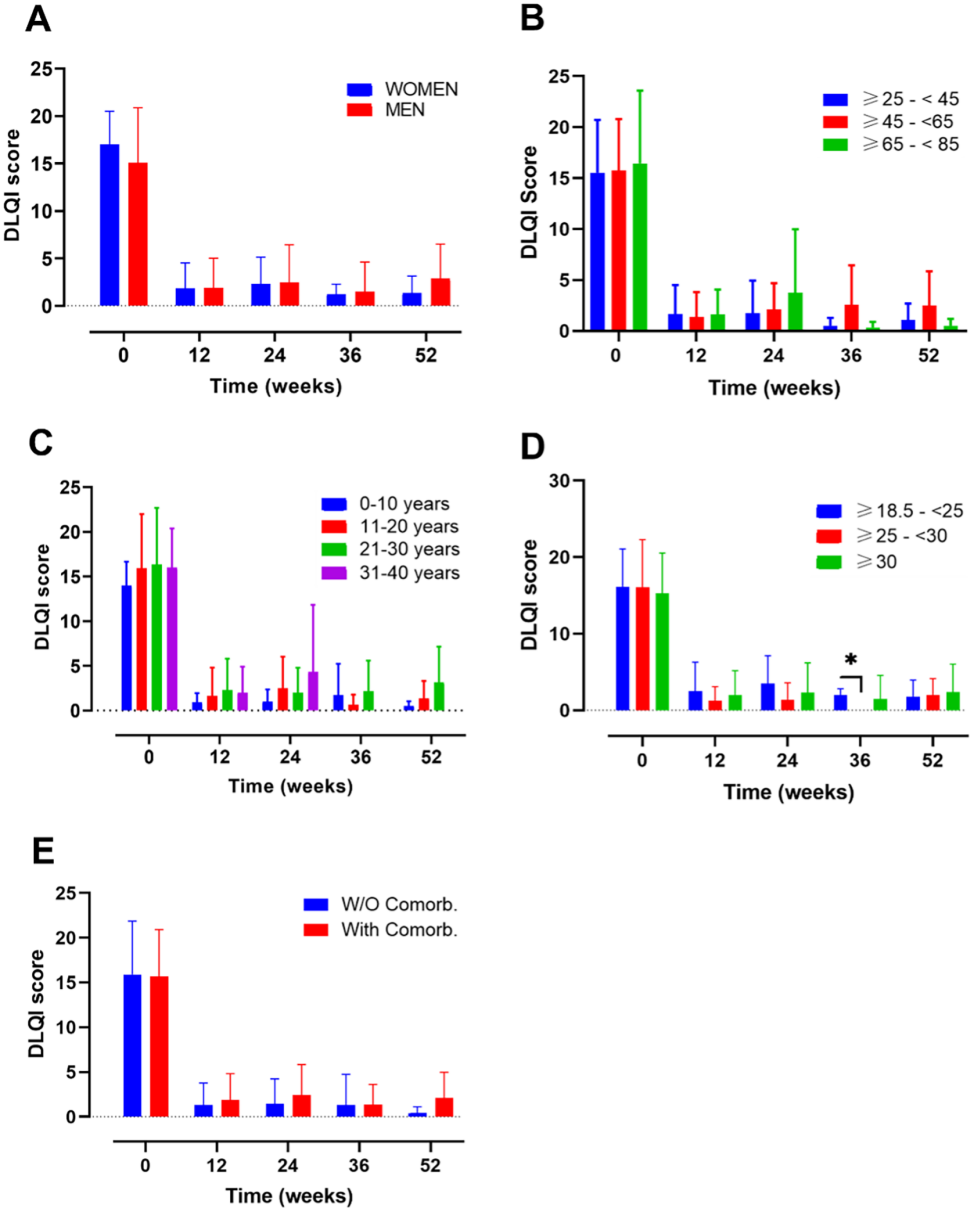
Evolution of dermatology life quality index (DLQI) score over time in different subgroups of patients during treatment with guselkumab. Evolution of DLQI score in A) female vs male; B) ranges of age; C) year of psoriasis evolution; D) ranges of body mass index and E) patients with or without comorbidities. Mixed-effects analysis, *, p<0.05. DLQI, dermatology life quality index.

Clinical efficacy was evaluated using the absolute PASI ^10^ score at baseline and weeks 4, 12, 24, 36, and 52 ( Figure 1). The impact of PSO during treatment was also assessed by the dermatology life quality index (DLQI) ^11^ at baseline and weeks 4, 12, 24, 36, and 52 ( Figure 2).

Several subgroups of patients were established to understand and compare the effectiveness, survival and safety of GUS in different profiles of patients found in real world evidence (RWE): 1) gender (female vs male); 2) ranges of age (≥25-<45, ≥45-<65, ≥65-<85); 3) years of PSO evolution (0-10, 11-20, 21-30, 31-40); 4) ranges of BMI (normal weight, overweight, obese) and 5) presence or absence of comorbidities.

Treatment survival was evaluated through Kaplan-Meyer survival curves according to the variables corresponding to baseline demographic characteristics, if any. Survival was evaluated up to week 93. For drug survival analysis, treatment discontinuations due to any cause were the event of interest.

Primary failure was considered failure to reach PASI 90 or PASI>3 after applying the biologics for 12 weeks, so secondary failure was defined as failure to maintain PASI 90 or PASI>3 after 12 weeks of treatment subsequently.

Safety was also evaluated according to treatment-emerging adverse events (AEs) and serious AEs that motivate discontinuation of treatment. Laboratory tests were performed according to individual clinical practice in every center.

### Statistical analysis

Categorical and quantitative variables are reported with frequencies (%), and with mean and standard deviation (SD), respectively. Differences between groups in PASI and DLQI, were calculated using a mixed-effects analysis. Survival was calculated using Kaplan-Meyer curves and log-rank tests. A p<0.05 was considered statistically significant. Data were analysed using GraphPad Prism version 8.0.0 for Windows (GraphPad Software, **RRID:** SCR_002798, San Diego, California USA.

## Results

### Patient demographic and clinical characteristics

A total of 87 patients were included. ^34^ Their demographic data, comorbidities, and baseline characteristics of disease at the start of GUS are summarized in Table 1. The population was composed of 60.9% male, with a mean age and PSO evolution of 49.9 (14.6) and 20.4 (9.5) years, respectively. On average their BMI was 29.22 (5.8) and they presented with multiple comorbidities such as: psoriatic arthritis (PSA, 13.8%), diabetes (20.7%), arterial hypertension (AHT, 23.0%), dyslipidaemia (28.7%), depression (13.8%) and fatty liver disease (8.0%). Their clinical characteristic scores were mean PASI 14.6 (7.2), mean BSA 22.3 (16.6), mean VAS pruritus 6.0 (2.2) and mean DLQI 15.8 (5.4). Of note, eighty-two patients included had been previously under biologic treatments.

**Table 1.** Demographic and clinical characteristics of the study population.

Patients (n=87)	
**Age (SD)**	49.9 (14.6)
**Years PSO Evolution (SD)**	20.4 (9.5)
**Gender, % (n)**	
Female	39.1 (34)
Male	60.9 (53)
**BMI, % (n)**	29.22 (5.8)
**Comorbidities, % (n)**	
Psoriatic Arthritis	13.8 (12)
Diabetes	20.7 (18)
AHT	23.0 (20)
Dyslipidaemia	28.7 (25)
Depression	13.8 (12)
Fat liver	8.0 (7)
**Score (SD)**	
PASI	14.6 (7.2)
BSA	22.3 (16.6)
VAS Pruritus	6.0 (2.2)
DLQI	15.8 (5.4)
**Previous Bio. Therapy, % (n)**	
0	5.7 (5)
1	26.4 (23)
2	18.4 (16)
≥3	49.4 (43)

AHT, arterial hypertension; BMI, body mass index; BSA, Body surface area; DLQI, dermatology life quality index; PASI, Psoriasis Area and Severity Index; PSO, Psoriasis; SD, standard deviation; VAS, visual analog scale.

### Effectiveness

Multiple subgroup analyses were performed to detect which variables could modify the response to GUS: gender, age, PSO duration, BMI, or presence of other comorbidities.

*Gender*

This analysis included a total of n=34 female and n=53 male ( Figure 1A). At baseline female and male presented with a PASI score of 14.23 (5.93) and 14.9 (8.00), respectively (Supplementary Table 1). After 12 weeks of treatment the values decreased to 1.43 (1.93) and 1.99 (2.42) for women and men. Also, at 52 weeks, PASI values remain low (female 0.36 (0.73) vs male 1.07 (1.23)) (Supplementary Table 1).

Mean DLQI values at baseline were 17.00 (3.51) for female and 15.09 (5.79) for male ( Figure 2A). After 12 weeks, DLQI decreased markedly to 1.85 (2.67) and 1.88 (3.15) for women and men, respectively (Supplementary Table 2). DLQI values also remain low after one year of treatment with a mean value of 1.38 and 2.88 for women and men respectively.

After 52 weeks of follow-up, no gender-related differences in PASI and DLQI scores were found ( Figures 1, 2).

*Ranges of age*

Another analysis was made by age subgroups where 85 participants were included ( Figure 1B). A group including patients under 25 years was not included in the analysis, due to small sample size (n=2). At baseline the mean PASI value for the groups ≥25-<45, ≥45-<65, ≥65-<85 was 14.18 (5.79), 13.72 (7.13) and 17.32 (8.90), respectively. After 12 weeks of treatment the mean value corresponding to this ranges of age ≥25-<45, ≥45-<65, ≥65-<85 was 1.70 (1.79), 1.15 (1.32) and 3.04 (3.80) respectively; and after 52 weeks these mean values remained low at 0.58 (0.96), 1.15 (1.25) and 1.05 (1.42) respectively (Supplementary Table 1).

The mean DLQI value presented with a similar trend showing at baseline values of 15.54 (5.18), 15.76 (5.04) and 16.45 (7.15) ( Figure 2B).

After 12 weeks of treatment, all the groups presented a mean DLQI score lower than 1.7 and after 52 weeks of treatments the mean DLQI values were 1.1 (1.59), 2.5 (3.38) and 0.5 (0.71) for the ranges of age ≥25-<45, ≥45-<65, ≥65-<85, respectively (Supplementary Table 2).

At 52 weeks of follow-up, neither PASI nor DLQI differences were noted among the subgroups mentioned at the timepoints evaluated.

*Years of PSO evolution*

An analysis based on the number of years of PSO evolution was also performed, including 16, 34, 27 and 10 patients in the following groups, respectively: 0-10, 11-20, 21-30, 31-40 ( Figure 1C, Figure 2C). Mean PASI values at base line were 12.68 (4.19), 16.23 (8.76), 12.67 (5.3), 17.69 (8.34) for the following groups, respectively, 0-10, 11-20, 21-30, 31-40. After 12 weeks, all mean values remained under a score of 2.2 and at 52-weeks of treatment mean PASI values were 0.77 (0.59), 0.23 (0.83), 1.75 (1.26) and 0.5 (0.71) for the 0-10, 11-20, 21-30, 31-40 ranges of age, respectively ( Figure 1C) (Supplementary Table 1).

The mean DLQI values, presented a similar trend as PASI ( Figure 2C). For the 0-10, 11-20, 21-30, 31-40 groups, baseline data were 14.00 (2.66), 15.93 (6.06), 16.35 (6.32), 16.00 (4.38) and subsequently, 52-weeks data were 0.50 (0.55), 1.36 (1.96), 3.17 (3.97) and 0.00 (0.00) respectively (Supplementary Table 2).

No differences on PASI and DLQI scores at 52 weeks were found on both analyses, except for worse PASI score at 52 weeks of follow-up in the subgroup of patients with PSO of 21-30 years’ evolution ( Figure 1C, p=0.0163 between group 11-20 and 21-30).

*Ranges of BMI*

The analysis by BMI categories ( Figures 1D, 2D), included 14, 28 and 41 patients in the BMI ranges normal-weight (18.5– 24.9), overweight (25–29.9) and obese (≥30), respectively ( Figure 1D). A total of 4 patients with a BMI <18.5, were excluded from the analysis due to the small sample size. At baseline, the overweight group presented the highest PASI score (*p=0.0187; overweight vs obese patients). After 12 weeks of treatment, the PASI values decreased to 1.17 (1.29), 2.33 (3.24) and 1.73 (1.75) for the normal weight, overweight and obese, respectively (Supplementary Table 1). No differences between groups were observed at this point. The data from the 3 groups presented a trend towards a decrease until week 52, were the PASI values were 0.44 (0.61) for normal-weight, 0.49 (0.90) for overweight and 1.48 (1.33) for obese patients. The DLQI values at baseline, presented a similar score for the 3 groups: normal-weight 16.14 (4.91), overweight 16.1 (6.19) and obese 15.3 (5.25) ( Figure 2). After the induction phase (12 weeks) DLQI values remain low and were maintained until 52 weeks of treatment: 1.75 (2.22) for normal-weight, 2.00 (2.16) for overweight and 2.38 (3.66) for obese patients (Supplementary Table 2).

No differences in PASI and DLQI were noted among the subgroups after 52 weeks of follow-up.

*Presence or absence of comorbidities*

Finally, subgroup analyses were also made according to the presence of baseline comorbidities including psoriatic arthritis (PSA), diabetes, dyslipidaemia, arterial hypertension, fatty liver disease, and depression ( Figures 1E, 2E). A total of 28 without comorbidities and 59 patients with comorbidities, were included in this study.

Patients with or without comorbidities presented a baseline mean PASI score of 14.29 (7.36) and 15.38 (7.02), respectively. After 12-weeks of treatment their mean PASI remained under 1.79 points and at the end of the study their values still decreased to 0.98 (1.19) and 0.58 (1.03), for patients with and without comorbidities, respectively (Supplementary Table 1).

On the other hand, the mean DLQI score at baseline was very similar between both groups (with comorbidities: 15.67 (5.23); without comorbidities: 15.92 (5.93)). At the end of the study these values decreased to 2.13 (2.87) and 0.38 (0.74), for patients with and without comorbidities, respectively (Supplementary Table 2).

There were no differences in PASI nor DLQI values for patients with and without comorbidities along the 52-weeks of study.

### Drug survival and safety

Drug survival was evaluated in every subgroup of patients up to week 93 of treatment, to study which variables could be associated with a greater or poorer treatment maintenance.

After 52 weeks of follow-up, no serious adverse events were detected. Only one adverse event (headache) and three discontinuations were notified (one primary failure and two secondary failures). No infections were reported.

*Gender*

**Figure 3.**
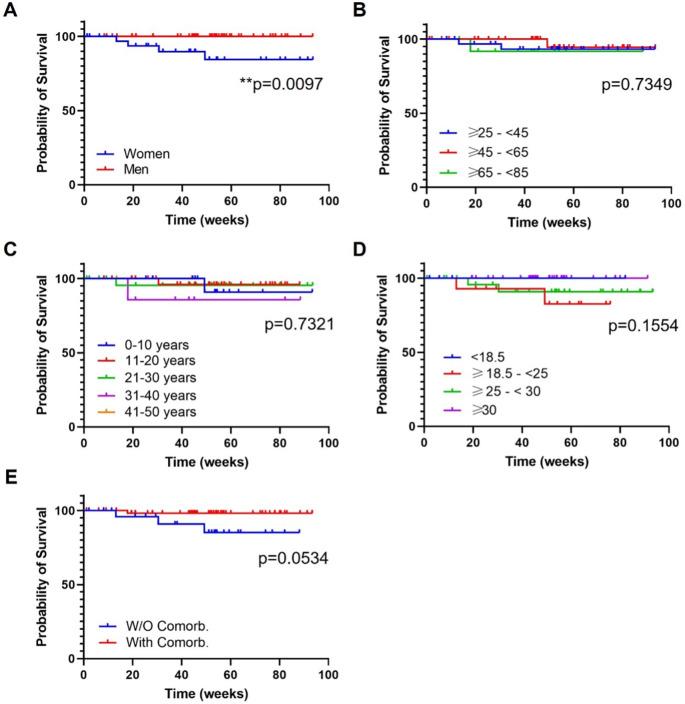
Treatment survival according to baseline demographic characteristics. A) Gender; B) Ranges of age; C) Years of psoriasis evolution; D) Ranges of body mass index; E) Presence or absence of comorbidities. Log-rank (Mantel Cox) test; *, p<0.01; **, p<0.001. W/O Comorb., without comorbidities; With Comorb., with comorbidities.

In this analysis, mean follow-up time was 46.2 weeks (25.2) for female and 49.8 weeks (22.2) for male. At 93 weeks of treatments the survival proportions were 84.4% for women and 100% for men. The log-rank test showed a statistically significant difference among both curves ( Figure 3A; p=0.0097). Four treatment discontinuations were reported, all in the female group: headache, primary failure, and two secondary failures.

*Ranges of age*

The following ranges of age, ≥25-<45, ≥45-<65, ≥65-<85, presented a mean follow up of 52.4 (21.8), 44.7 (24.9) and 44.6 (21.4) weeks, respectively. At 93 weeks of treatment, survival proportions were 93.2% (group ≥25-<45), 94.4% (group ≥45-<65) and 91.7% (group ≥65-<85) ( Figure 3B). There was no difference in drug survival between groups ( Figure 3B; non-significant (ns), p=0.7349). There were two discontinuations in the ≥25-<45 group (headache, secondary failure), one in the ≥45-<65 group (secondary failure) and one in the ≥65-<85 group (primary failure).

*Years of PSO evolution*

The analysis of the four subgroups of patients attending to ranges of PSO evolution showed a mean follow-up of (weeks): 53.4 (15.3) (range 0-10 years), 49.7 (23.1) (range 11-20 years), 46.6 (26.4) (range 21-30 years) and 47.8 (25.6) (range 31-40 years). Maximum follow up for all groups were 88 weeks. At this point, the survival proportions were: 90.9% (range 0-10 years), 96% (range 11-20 years), 95.5% (range 21-30 years) and 85.7 (range 31-40 years). There was no difference in drug survival between groups ( Figure 3C; ns, p=0.7321) and discontinuations were observed in all groups: two secondary failure (range 0-10 and 11-20 years), one headache (range 21-30 years) and one primary failure (range 31-40 years).

*Ranges of BMI*

Mean follow-up time for normal weight, overweight and obese patients was 46.6 (20.1), 49.6 (28.5) and 50.5 (17.7), respectively. The maximum common follow-up was 76 weeks, at this timepoint where the survival proportions were: 82.5% (normal weight), 90,9% (overweight) and 100% (obese). There was no difference in drug survival between groups ( Figure 3D; ns, p=0.1554). There were two discontinuations in the overweight group (headache, secondary failure) and another two in the overweight group (primary and secondary failure).

*Presence or absence of comorbidities*

Mean follow-up for patients with and without comorbidities was 50.6 (23.1) and 44.2 (23.6), respectively. After 88 weeks of treatment (maximum follow-up point between both groups) the survival proportions were 98,1% and 85,1% for patients with and without comorbidities, respectively. There was a trend towards a statistical significance difference between both groups ( Figure 3E; ns, p=0.0534). A total of four discontinuations were reported: one in the group with comorbidities (primary failure) and three in the group without comorbidities (headache, two secondary failures).

### Study limitations

Some limitations of this study are its nature of retrospective study and the presence of unbalanced groups and subsequently the absence of adjustment in clinical and demographic basal characteristics between them. Some groups presented a limited sample size. Also, safety evaluation was suboptimal in comparison to clinical trials, due to the clinical practice setting of the study (no local inflammatory reactions have been described at the injection site, nor upper respiratory tract infections).

## Discussion

Almost five years after the approval of GUS for moderate-to-severe PSO in patients candidates for systemic therapy, long term responses to GUS in open label extensions of RCTs and clinical practice series have been published. ^12^^–^^14^

In 2018, Gordon and colleagues published a pooled analysis of phase III VOYAGE 1 and 2 where they evaluated the efficacy of GUS vs placebo and adalimumab attending to demographic and baseline clinical characteristics. ^4^ They analysed a total of 1829 patients in which they evaluated the percentage of patients achieving investigator's global assessment (IGA) 01/1 and IGA 0 in the study. Their conclusions were that GUS was superior to placebo at week 16 and to adalimumab at 24 weeks of treatment among different subgroups of patients (baseline demographics, disease characteristics and previous PSO treatments). Also, GUS superiority to adalimumab, evaluated by the proportion of patients achieving IGA 0/1, presented a similar response between male and female (83.9% vs 83.5%), baseline ranges of age (<45, ≥45-<65, ≥65: 87.0% vs 80.1% vs 80.5%, respectively), PSO duration (years) (<15 vs ≥15; 83.7% vs 83.8%), previous systemic and biologic treatments among others. Specifically for obese patients, GUS presented better performance than the anti-TNFα. ^12^ To our knowledge, no DLQI comparisons have been performed with GUS in subgroups of patients. Our data indicate that demographic data do not contribute any impact on DLQI outcomes over time.

When evaluating RWE studies, indirect comparison is complicated as data is plotted in different formats and diverse clinical parameter are used to determine PSO improvement. Despite of that, by evaluating the effectiveness of GUS through absolute PASI, our data presented similar overall results. The effectiveness of GUS seems not to be impacted by gender, age, PSO duration, BMI nor by presence or absence of comorbidities over time. However, it is worth highlighting than older patients (≥65-<85) and those with longer story of PSO tended to present poorer responses than their counterparts. Also, it has to me mentioned that the lack of significant differences could be influenced at this point by a small sample size.

BMI data analysis in previous reports has yielded contradictory results; in some studies a worse response has been observed in obese patients, ^15^^–^^34^ whereas in others, no differences in response have been found among BMI subgroups. ^11^^,^^23^ Snast *et al.* observed a PASI90 response at week 24 in 23% of obese patients, versus 56% in non-obese participants, but statistical significance was not reached (p=0.07). ^25^ Galluzzo *et al.* did not find obesity to be associated with poorer response. ^23^ In our study, we also observed no response differences were detected among BMI subgroups attending to absolute PASI or DLQI scores. According to another study, patients with comorbidities have poorer responses ^23^; PASI100 response rate was 100% for patients without comorbidities, in contrast to 46.2% in participants with comorbidities. ^23^ Our study detected no response differences among comorbidities and without comorbidities subgroups.

In general, our study showed that drug survival did not differ among subgroups defined by age, BMI, year of PSO evolution and comorbidities but was better in men vs women [Log-rank (Mantel Cox) test, p=0.0097] ( Figure 3A). According to Iznardo *et al*, ^25^ worse drug survival was seen in patients with psoriatic arthritis; however, we didn’t evaluate this comorbidity independently.

## Conclusions

Our research included a total of 87 patients treated with GUS under real world conditions. Our main goal was to evaluate the consistency of GUS efficacy across patient subgroups. Ultimately, no baseline features affected the efficacy of GUS, including sex, age, PSO duration, BMI or comorbidities. Drug survival was only affected by the gender of the patient, with worse outcomes for females (survival proportions at 93 weeks: 84.4% and 100% for women and men (p=0.0097), respectively). Our results indicate that GUS is a very versatile biologic drug for the treatment of different profiles of patients found in a real practice setting, showing effectiveness, safety, and a favorable persistence performance and also are consistent with those of previous studies that demonstrated the effectiveness of GUS in non-RCT conditions.

## Data availability

### Underlying data

Figshare: Effectiveness, survival and safety of guselkumab attending to basal characteristics in moderate-to-severe psoriatic patients: a cohort study. https://doi.org/10.6084/m9.figshare.20981053.v1. ^34^

This project contains the following underlying data.

- Data file 1. Demographics.csv
- Data file 2. Baseline data.csv
- Data file 3. Data at 12 weeks of treatment.csv
- Data file 4. Data at 24 weeks of treatment.csv
- Data file 5. Data at 36 weeks of treatment.csv
- Data file 6. Data at 52 weeks of treatment.csv

Data are available under the terms of the Creative Commons Zero “No rights reserved” data waiver (CC0 1.0 Public domain dedication).

## Authors contributions

**Conceptualization:** RRV, LRFF, JCAH, APP, FVC, MGG.

**Resources:** RRV, LRFF, MGG, APG, JCAH.

**Writing – Original Draft Preparation:** RRV.

**Writing – Review & Editing:** RRV, LRFF, JCAH, APP, FVC, MGG.
