# Supplementary material for: Effectiveness, survival and safety of guselkumab attending to basal characteristics in moderate-to-severe psoriatic patients: a cohort study
Source: F1000Res. 2022 Nov 7;11:1178. Originally published 2022 Oct 17. [Version 2] doi: 10.12688/f1000research.122945.2 (PMC9755754; doi:10.12688/f1000research.122945.2)
Supplement: Peer review history

# Peer review: Reviewer response for version 2

**DOI:** 10.5256/f1000research.140623.r154409

**Published:** 2022-12-15

**Authors:** Jose Manuel Carrascosa

**Affiliations:** 1 Dermatology Department, Hospital Universitari Germans Trias i Pujol, Badalona, Spain

The authors present a manuscript based on a series of patients treated with guselkumab in a single center. The authors evaluate the differences in response and quality of life based on various parameters related to the epidemiological and clinical background. The authors only find a relationship between a worse response and female gender. Although the elderly patients numerically showed worse response parameters. This is a well-written article that may be useful to clinicians in decision-making in biological therapy. However, some comments should be noted:

- In Figures 1 and 2 (B), indicate “years” in the intervals of ranges of age, to facilitate understanding.
- In the abbreviation of "without", look for an alternative to W/O which, in dermatological reading, is usually identified with "water/oil" and can lead to confusion.
- The authors must identify as a limitation the fact that they include all the comorbidities in a single variable, when they are very different from each other, with a very different contribution to the therapeutic response. The affirmations made in relation to the absence of impact of comorbidities must be prudent.
- The authors should expand somewhat more on the reflection on the worse response observed in women, which has been observed in the literature. ^1^ Since, in fact, it is the only relationship they found to condition clinical response.

Are enough details provided of any physical examination and diagnostic tests, treatment given and outcomes?

Yes

Is the background of the cases’ history and progression described in sufficient detail?

Yes

Is sufficient discussion included of the importance of the findings and their relevance to future understanding of disease processes, diagnosis or treatment?

Yes

Is the conclusion balanced and justified on the basis of the findings?

Yes

Reviewer Expertise:

Inflammatory skin diseases (psoriasis, atopic dermatitis, eczema, photobiology)

I confirm that I have read this submission and believe that I have an appropriate level of expertise to confirm that it is of an acceptable scientific standard.

## References

[1] : Biologic drug survival rates in the era of anti-interleukin-17 antibodies: a time-period-adjusted registry analysis. *Br J Dermatol* .184(6) : 10.1111/bjd.19701 1094-1105 10.1111/bjd.19701 33289075PMC8248155

# Peer review: Reviewer response for version 2

**DOI:** 10.5256/f1000research.140623.r155169

**Published:** 2022-11-16

**Authors:** Pedro Mendes Bastos

**Affiliations:** 1 Dermatology Center, Hospital CUF Descobertas, Lisboa, Portugal

The corrections have addressed all the points.

Are enough details provided of any physical examination and diagnostic tests, treatment given and outcomes?

Yes

Is the background of the cases’ history and progression described in sufficient detail?

Yes

Is sufficient discussion included of the importance of the findings and their relevance to future understanding of disease processes, diagnosis or treatment?

Yes

Is the conclusion balanced and justified on the basis of the findings?

Yes

Reviewer Expertise:

NA

I confirm that I have read this submission and believe that I have an appropriate level of expertise to confirm that it is of an acceptable scientific standard.

# Peer review: Reviewer response for version 1

**DOI:** 10.5256/f1000research.134999.r153465

**Published:** 2022-11-01

**Authors:** Pedro Mendes Bastos

**Affiliations:** 1 Dermatology Center, Hospital CUF Descobertas, Lisboa, Portugal

I consider this study to be of high value in order to gain greater insight into guselkumab’s behaviour as a psoriasis treatment in the real world setting. It brings valuable information and the conclusions are soundly based on the results; however, minor revisions are needed. These revisions are listed below.

1. *"It impacts the general quality of life (usually measured by the dermatology life quality index (DLQI)) and the pruritus that may cause (visual analog scale (VAS) pruritus) and influences the psychological sphere, work productivity as well as personal and family relationships.” - *This sentence needs rewriting because it is not clear.
2. *"anti-interleukin (IL)-173,4 and anti-IL23p19.” *- Antagonists at the end?
3. *"Our current understanding of the pathophysiology of PSO..." *- It’s better to replace " *has been driven*” by “ ***is characterized by **a cytokine disbalance with predominant involvement of the interleukin (IL)-23/Th-17 axis, as well as keratinocyte hyperproliferation and immune activation*”.
4. *"and **was **confirmed by their therapeutic success".* The “was” is missing.
5. Correct the sentence, according to this suggestion: *"Furthermore, GUS treatment has been compared to other biologics, such as secukinumab, and the CLEAR head-to-head trial proved the superiority of this anti-IL23p19 antagonist."*
6. The sentence: *"GUS was superior to placebo at week 16 and superior to adalimumab at week 24 in all subpopulations except in the Black or African American population, which included few patients”* is not clear. ADA was superior to GUS in the Black or African American population? This is not clear. Please rewrite the sentence.
7. *"Kaplan-Meyer"* needs uniformization across the manuscript as it is spelled differently. Please uniformize.
8. Please correct: " *As a limitation of the study, we did not applied any method to avoid bias.*” - " *we did not apply*" would be the grammatically correct form.
9. In the methods section, you need to define what “ *moderate-to-severe psoriasis*” meant when recruiting. Was it just a question of PASI>10? Was it PASI>10 or DLQI>10? Was it other criteria? Please provide a sentence to clarify.
10. Primary failure was considered a failure to reach PASI 90 or PASI>3 after applying the biologics and secondary failure is defined as failure to maintain PASI 90 or PASI>3 after 12 weeks of treatment. - This sentence is confusing and needs clarification. What was the timeline for primary failure evaluation? PASI<3, right? Please rewrite the sentence.
11. " *Safety was also evaluated attending to treatment-emerging adverse events (AEs), serious AEs and discontinuations due to AEs and/or lack of efficacy (primary or secondary failure).*“ " *attending*" —> change to " *according*", please. I don’t understand how safety is assessed through a lack of efficacy.
12. " *No laboratory tests were performed*” - Not at all, or the performed laboratory tests were not considered for analysis in this study? Please clarify.
13. " *fat liver*" —> Please change to " *fatty liver disease*".
14. " *Their clinical characteristic scores were PASI 14.6 (7.2), BSA 22.3 (16.6), VAS pruritus 6.0 (2.2) and DLQI 15.8 (5.4)*” - Do you mean their mean characteristics scores? Please add “mean”.
15. " *DLQI values at baseline were 17.00 (3.51) for female and 15.09 (5.79) for male (Figure 2A). After 12 weeks, DLQI decreased markedly to 1.85 (2.67) and 1.88 (3.15) for women and men, respectively (Supplementary Table 2). DLQI values also remain low after one year of treatment*” - It looks as if the last sentence is missing the mean information for DLQI at 52 weeks. Could you include it?
16. " *At baseline the PASI value for the groups ≥25-<45, ≥45-<65, ≥65-<85 was 14.18 (5.79), 13.72 (7.13) and 17.32 (8.90), respectively.*“ - Do you mean their means scores? Please add “mean”.
17. " *After 12 weeks of treatment the value corresponding to this ranges of age ≥25-<45, ≥45-<65, ≥65-<85 was 1.70 (1.79), 1.15 (1.32) and 3.04 (3.80) respectively; and after 52 weeks these values remained low at 0.58 (0.96), 1.15 (1.25) and 1.05 (1.42) respectively (Supplementary Table 1). - *Do you mean their means scores? Please add “mean”.
18. " *The DLQI value presented with a similar trend showing at baseline values of 15.54 (5.18), 15.76 (5.04) and 16.45 (7.15) (Figure 2B). After 12 weeks of treatment, all the groups presented a DLQI score lower than 1.7 and after 52 weeks of treatments the DLQI values were 1.1 (1.59), 2.5 (3.38) and 0.5 (0.71) for the ranges of age ≥25-<45, ≥45-<65, ≥65-<85, respectively (Supplementary Table 2).*”- Do you mean their means scores? Please add “mean”.
19. " *PASI values at base line were 12.68 (4.19), 16.23 (8.76), 12.67 (5.3), 17.69 (8.34) for the following groups, respectively, 0-10, 11-20, 21-30, 31-40. After 12 weeks, all values remained under a score of 2.2 and at 52-weeks of treatment PASI values were 0.77 (0.59), 0.23 (0.83), 1.75 (1.26) and 0.5 (0.71) for the 0-10, 11-20, 21-30, 31-40 ranges of age, respectively (Figure 1C) (Supplementary Table 1)*.” - Do you mean their means scores? Please add “mean”.
20. " *The DLQI values, presented a similar trend as PASI’s*“ —> remove the ’s, please.
21. *"After 12 weeks of treatment, the PASI values decreased to 1.17 (1.29), 2.33 (3.24) and 1.73 (1.75) for the normal weight, overweight and obese, respectively (Supplementary Table 1)."* - Do you mean their means scores? Please add “mean”.
22. " *The data from the 3 groups presented a trend towards a decrease until week 52, were the PASI values were 0.44 (0.61) for normal-weight, 0.49 (0.90) for overweight and 1.48 (1.33) for obese patients. The DLQI values at baseline, presented a similar score for the 3 groups: normal-weight 16.14 (4.91), overweight 16.1 (6.19) and obese 15.3 (5.25) (Figure 2). After the induction phase (12 weeks) DLQI values remain low and were maintained until 52 weeks of treatment: 1.75 (2.22) for normal-weight, 2.00 (2.16) for overweight and 2.38 (3.66) for obese patients (Supplementary Table 2)*.” - Do you mean their means scores? Please add “mean”.
  " *were the PASI values were 0.44* “ —> This part is not clear. Please correct the language.
23. " *A total of 28 without comorbidities and 59 patients with comorbidities, were included in this study.*” - Remove the comma.
24. *"Patients with or without comorbidities presented a baseline PASI score of 14.29 (7.36) and 15.38 (7.02), respectively. After 12-weeks of treatment their PASI remained under 1.79 points and at the end of the study their values still decreased to 0.98 (1.19) and 0.58 (1.03), for patients with and without comorbidities, respectively (Supplementary Table 1)." -* Do you mean their means scores? Please add “mean”.
25. " *Drug survival was evaluated in every subgroup of patients up to week 93 of treatment, to study which variables could be associated with a greater or poorer treatment maintenance*.” - Remove the comma.
26. " *In this analysis, mean follow-up time was 46.2 (25.2) for female and 49.8 (22.2) for male.“ -* Do you mean weeks? Please add “weeks”.
27. " *Almost five years after the approval of GUS for moderate-to-severe PSO in patients candidates for systemic therapy, long term responses to GUS in open label extensions of RCTs and clinical practice series regarding have been published*.” - Please remove “regarding”.
28. " *Even though, our data, that evaluated the effectiveness of GUS through absolute PASI, presented similar overall results.*” - Please correct the language. I suggest: “ *Despite of that, by evaluating the effectiveness of GUS through absolute PASI, our data presented similar overall results*”.
29. " *Our results indicated that GUS is a very versatile biologic alternative, effective, secure with a good persistence performance, for the treatment of different profile of patients found in a real practice setting.*“ - This sentence needs language correction. I suggest: " *Our results indicate that GUS is a very versatile biologic drug for the treatment of different profiles of patients found in a real practice setting, showing effectiveness, safety, and a favorable persistence performance.*”
30. "Acknowledgments: The writing of this article was supported by a medical writer." - Could you provide the name of the medical writer or the company for which he/she works? It would be more transparent.

I would appreciate if all the corrections are sent to me separately or highlighted in the text for easier revision. Thank you.

Are enough details provided of any physical examination and diagnostic tests, treatment given and outcomes?

Yes

Is the background of the cases’ history and progression described in sufficient detail?

Yes

Is sufficient discussion included of the importance of the findings and their relevance to future understanding of disease processes, diagnosis or treatment?

Yes

Is the conclusion balanced and justified on the basis of the findings?

Yes

Reviewer Expertise:

NA

I confirm that I have read this submission and believe that I have an appropriate level of expertise to confirm that it is of an acceptable scientific standard, however I have significant reservations, as outlined above.

# Response

**Published:** 2022-11-01

**Authors:** Ricardo Ruiz Villaverde

**Affiliations:** Hospital Universitario San Cecilio, Spain

Response to reviewer:

 To Dr. Mendes Bastos,

 I consider this study to be of high value in order to gain greater insight into guselkumab’s behaviour as a psoriasis treatment in the real world setting. It brings valuable information and the conclusions are soundly based on the results; however, minor revisions are needed. These revisions are listed below.

**AUTHOR REPLY: Thank you very much for the exhaustive review you have carried out on our manuscript which will allow us to improve its quality.**

1. *"It impacts the general quality of life (usually measured by the dermatology life quality index (DLQI)) and the pruritus that may cause (visual analog scale (VAS) pruritus) and influences the psychological sphere, work productivity as well as personal and family relationships.” - *This sentence needs rewriting because it is not clear. **AUTHOR REPLY: It has been rephrasing according to the reviewer's suggestion.**
2. *"anti-interleukin (IL)-173,4 and anti-IL23p19.” *- Antagonists at the end? **AUTHOR REPLY: Selective blockers.**
3. *"Our current understanding of the pathophysiology of PSO..." *- It’s better to replace " *has been driven*” by “ ***is characterized by*** *a cytokine disbalance with predominant involvement of the interleukin (IL)-23/Th-17 axis, as well as keratinocyte hyperproliferation and immune activation*”. **AUTHOR REPLY: It has been rephrasing according to the reviewer's suggestion.**
4. *"and **was **confirmed by their therapeutic success".* The “was” is missing. **AUTHOR REPLY. It has been added.**
5. Correct the sentence, according to this suggestion: *"Furthermore, GUS treatment has been compared to other biologics, such as secukinumab, and the CLEAR head-to-head trial proved the superiority of this anti-IL23p19 antagonist."* **AUTHOR REPLY: It has been rephrased according to the reviewer's suggestion.**
6. The sentence: *"GUS was superior to placebo at week 16 and superior to adalimumab at week 24 in all subpopulations except in the Black or African American population, which included few patients”* is not clear. ADA was superior to GUS in the Black or African American population? This is not clear. Please rewrite the sentence. **AUTHOR REPLY: It has been rephrased according to the reviewer's suggestion.**
7. *"Kaplan-Meyer"* needs uniformization across the manuscript as it is spelled differently. Please uniformize. **AUTHOR REPLY: It has been reviewed. We totally agreed with the reviewer. **
8. Please correct: " *As a limitation of the study, we did not applied any method to avoid bias.*” - " *we did not apply*" would be the grammatically correct form. **AUTHOR REPLY: It has been reviewed. We totally agreed with the reviewer. **
9. In the methods section, you need to define what “ *moderate-to-severe psoriasis*” meant when recruiting. Was it just a question of PASI>10? Was it PASI>10 or DLQI>10? Was it other criteria? Please provide a sentence to clarify. **AUTHOR REPLY: It has been reviewed and all the criteria have been added.**
10. Primary failure was considered a failure to reach PASI 90 or PASI>3 after applying the biologics and secondary failure is defined as failure to maintain PASI 90 or PASI>3 after 12 weeks of treatment. - This sentence is confusing and needs clarification. What was the timeline for primary failure evaluation? PASI<3, right? Please rewrite the sentence. **AUTHOR REPLY: It has been rephrased according to the reviewer's suggestion.**
11. " *Safety was also evaluated attending to treatment-emerging adverse events (AEs), serious AEs and discontinuations due to AEs and/or lack of efficacy (primary or secondary failure).*“ " *attending*" —> change to " *according*", please. I don’t understand how safety is assessed through a lack of efficacy. **AUTHOR REPLY: It has been rephrased according to the reviewer's suggestion**
12. " *No laboratory tests were performed*” - Not at all, or the performed laboratory tests were not considered for analysis in this study? Please clarify. **AUTHOR REPLY: It has been reviewed and clarified.**
13. " *fat liver*" —> Please change to " *fatty liver disease*". **AUTHOR REPLY: It has been rephrased according to the reviewer's suggestion.**
14. " *Their clinical characteristic scores were PASI 14.6 (7.2), BSA 22.3 (16.6), VAS pruritus 6.0 (2.2) and DLQI 15.8 (5.4)*” - Do you mean their mean characteristics scores? Please add “mean”. **AUTHOR REPLY: It has been rephrased according to the reviewer's suggestion.**
15. " *DLQI values at baseline were 17.00 (3.51) for female and 15.09 (5.79) for male (Figure 2A). After 12 weeks, DLQI decreased markedly to 1.85 (2.67) and 1.88 (3.15) for women and men, respectively (Supplementary Table 2). DLQI values also remain low after one year of treatment*” - It looks as if the last sentence is missing the mean information for DLQI at 52 weeks. Could you include it? **AUTHOR REPLY: The values have been added.**
16. " *At baseline the PASI value for the groups ≥25-<45, ≥45-<65, ≥65-<85 was 14.18 (5.79), 13.72 (7.13) and 17.32 (8.90), respectively.*“ - Do you mean their means scores? Please add “mean”. **AUTHOR REPLY: It has been rephrased according to the reviewer's suggestion.**
17. " *After 12 weeks of treatment* *the value corresponding to this ranges of age ≥25-<45, ≥45-<65, ≥65-<85 was 1.70 (1.79), 1.15 (1.32) and 3.04 (3.80) respectively; and after 52 weeks these values remained low at 0.58 (0.96), 1.15 (1.25) and 1.05 (1.42) respectively (Supplementary Table 1). - *Do you mean their means scores? Please add “mean”. **AUTHOR REPLY: It has been rephrased according to the reviewer's suggestion.**
18. " *The DLQI value presented with a similar trend showing at baseline values of 15.54 (5.18), 15.76 (5.04) and 16.45 (7.15) (Figure 2B). After 12 weeks of treatment, all the groups presented a DLQI score lower than 1.7 and after 52 weeks of treatments the DLQI values were 1.1 (1.59), 2.5 (3.38) and 0.5 (0.71) for the ranges of age ≥25-<45, ≥45-<65, ≥65-<85, respectively (Supplementary Table 2).*”- Do you mean their means scores? Please add “mean”. **AUTHOR REPLY: It has been rephrased according to the reviewer's suggestion.**
19. " *PASI values at base line were 12.68 (4.19), 16.23 (8.76), 12.67 (5.3), 17.69 (8.34) for the following groups, respectively, 0-10, 11-20, 21-30, 31-40. After 12 weeks, all values remained under a score of 2.2 and at 52-weeks of treatment PASI values were 0.77 (0.59), 0.23 (0.83), 1.75 (1.26) and 0.5 (0.71) for the 0-10, 11-20, 21-30, 31-40 ranges of age, respectively (Figure 1C) (Supplementary Table 1)*.” - Do you mean their means scores? Please add “mean”. **AUTHOR REPLY: It has been rephrased according to the reviewer's suggestion.**
20. " *The DLQI values, presented a similar trend as PASI’s*“ —> remove the ’s, please. **AUTHOR REPLY: It has been removed.**
21. *"After 12 weeks of treatment, the PASI values decreased to 1.17 (1.29), 2.33 (3.24) and 1.73 (1.75) for the normal weight, overweight and obese, respectively (Supplementary Table 1)."* - Do you mean their means scores? Please add “mean”. **AUTHOR REPLY: It has been rephrased according to the reviewer's suggestion.**
22. " *The data from the 3 groups presented a trend towards a decrease until week 52, were the PASI values were 0.44 (0.61) for normal-weight, 0.49 (0.90) for overweight and 1.48 (1.33) for obese patients. The DLQI values at baseline, presented a similar score for the 3 groups: normal-weight 16.14 (4.91), overweight 16.1 (6.19) and obese 15.3 (5.25) (Figure 2). After the induction phase (12 weeks) DLQI values remain low and were maintained until 52 weeks of treatment: 1.75 (2.22) for normal-weight, 2.00 (2.16) for overweight and 2.38 (3.66) for obese patients (Supplementary Table 2)*.” - Do you mean their means scores? Please add “mean”. **AUTHOR REPLY: It has been rephrased according to the reviewer's suggestion.**
  " *were the PASI values were 0.44* “ —> This part is not clear. Please correct the language. **AUTHOR REPLY: It has been rephrased according to the reviewer's suggestion.**
23. " *A total of 28 without comorbidities and 59 patients with comorbidities, were included in this study.*” - Remove the comma. **AUTHOR REPLY: It has been removed.**
24. *"Patients with or without comorbidities presented a baseline PASI score of 14.29 (7.36) and 15.38 (7.02), respectively. After 12-weeks of treatment their PASI remained under 1.79 points and at the end of the study their values still decreased to 0.98 (1.19) and 0.58 (1.03), for patients with and without comorbidities, respectively (Supplementary Table 1)." -* Do you mean their means scores? Please add “mean”. **AUTHOR REPLY: It has been rephrased according to the reviewer's suggestion.**
25. " *Drug survival was evaluated in every subgroup of patients up to week 93 of treatment, to study which variables could be associated with a greater or poorer treatment maintenance*.” - Remove the comma. **AUTHOR REPLY: It has been removed.**
26. " *In this analysis, mean follow-up time was 46.2 (25.2) for female and 49.8 (22.2) for male.“ -* Do you mean weeks? Please add “weeks”. **AUTHOR REPLY: It has been rephrased according to the reviewer's suggestion.**
27. " *Almost five years after the approval of GUS for moderate-to-severe PSO in patients candidates for systemic therapy, long term responses to GUS in open label extensions of RCTs and clinical practice series regarding have been published*.” - Please remove “regarding”. **AUTHOR REPLY: It has been rephrased according to the reviewer's suggestion.**
28. " *Even though, our data, that evaluated the effectiveness of GUS through absolute PASI, presented similar overall results.*” - Please correct the language. I suggest: “ *Despite of that, by evaluating the effectiveness of GUS through absolute PASI, our data presented similar overall results*”. **AUTHOR REPLY: It has been rephrased according to the reviewer's suggestion.**
29. " *Our results indicated that GUS is a very versatile biologic alternative, effective, secure with a good persistence performance, for the treatment of different profile of patients found in a real practice setting.*“ - This sentence needs language correction. I suggest: " *Our results indicate that GUS is a very versatile biologic drug for the treatment of different profiles of patients found in a real practice setting, showing effectiveness, safety, and a favorable persistence performance* *.*” **AUTHOR REPLY: It has been rephrased according to the reviewer's suggestion.**
30. "Acknowledgments: The writing of this article was supported by a medical writer." - Could you provide the name of the medical writer or the company for which he/she works? It would be more transparent. **AUTHOR REPLY: It has been added.**
